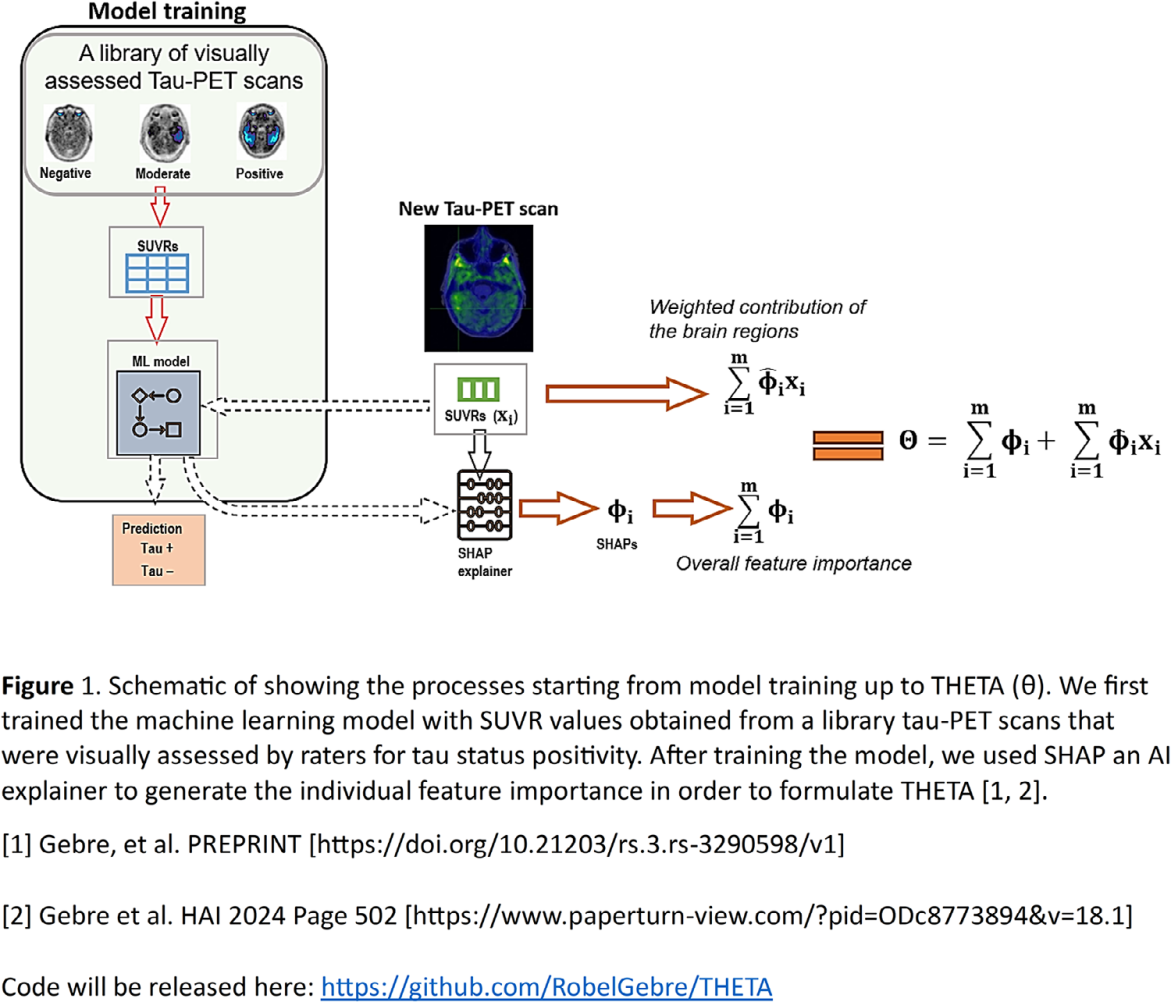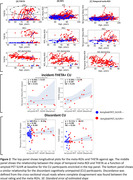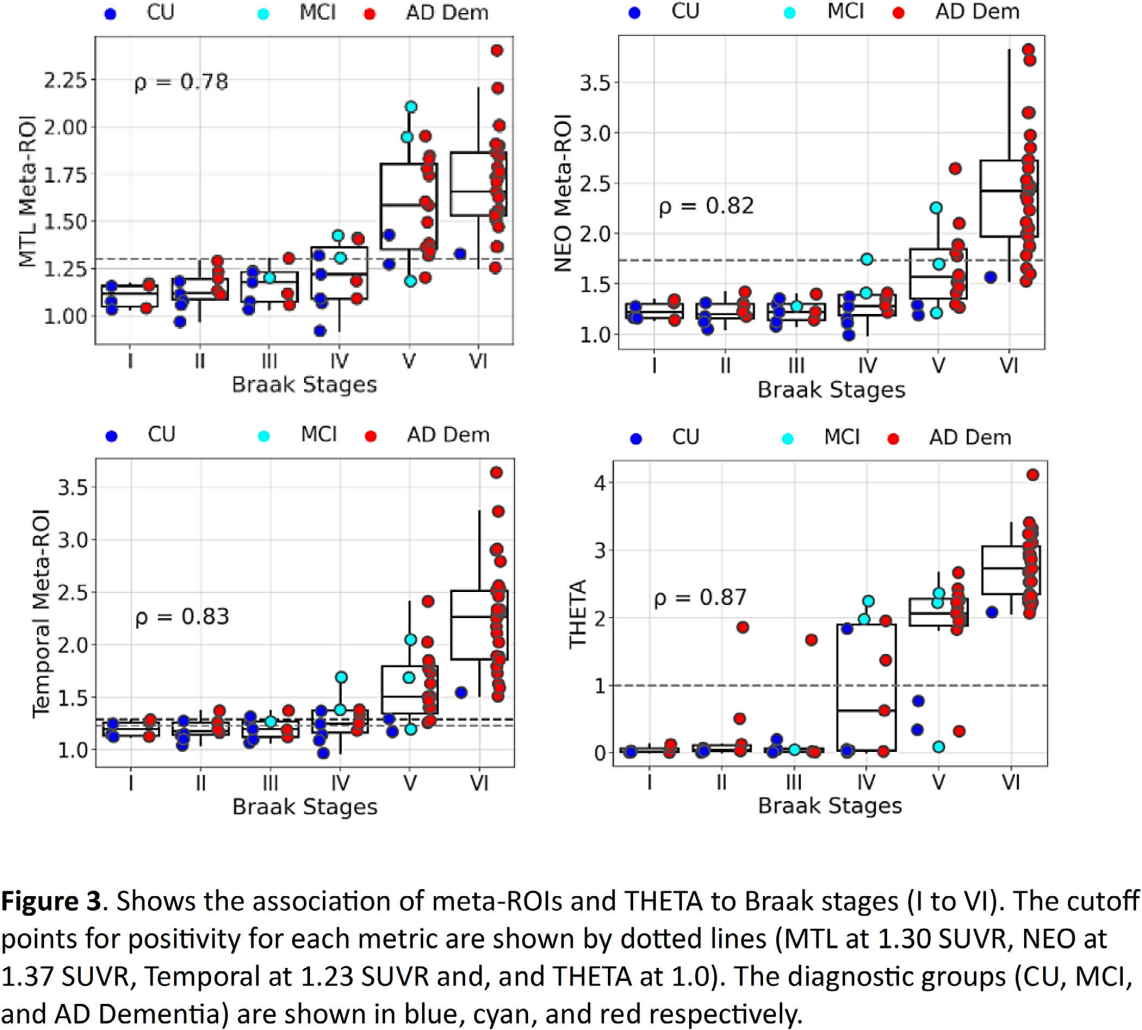# Validation Of the Tau Heterogeneity Evaluation in Alzheimer’s Disease (THETA) Score Using Longitudinal and Histopathology Data

**DOI:** 10.1002/alz.091770

**Published:** 2025-01-03

**Authors:** Robel K Gebre, Alexis Moscoso, Sheelakumari Raghavan, Heather J. Wiste, Fiona Heeman, Alejandro Costoya‐Sánchez, Christopher G. Schwarz, Anthony J. Spychalla, Val J. Lowe, Jonathan Graff‐Radford, David S. Knopman, Ronald C. Petersen, Michael Schöll, Melissa E. Murray, Clifford R. Jack, Prashanthi Vemuri

**Affiliations:** ^1^ Mayo Clinic, Rochester, MN USA; ^2^ Wallenberg Centre for Molecular and Translational Medicine, University of Gothenburg, Gothenburg Sweden; ^3^ Department of Psychiatry and Neurochemistry, Institute of Neuroscience and Physiology, University of Gothenburg, Mölndal Sweden; ^4^ University of Gothenburg, Gothenburg Sweden; ^5^ University of Santiago de Compostela, Santiago de Compostela Spain; ^6^ Department of Radiology, Mayo Clinic, Rochester, MN USA; ^7^ Department of Neurology, Mayo Clinic, Rochester, MN USA; ^8^ Mayo Clinic, Jacksonville, FL USA

## Abstract

**Background:**

We recently developed a novel tau‐PET summary measure THETA, capturing regional heterogeneity and identifying tau status, using ground truth visual assessments from a large single‐center cross‐sectional dataset and validated on independent cohorts [1, 2]. In this study, we aimed to evaluate the performance of THETA on longitudinal and histopathology data.

**Method:**

We included longitudinal tau‐PET ([^18^F]flortaucipir) data from 696 Mayo Clinic Study of Aging (MCSA) and ADRC participants, with histopathology in n = 90. Fig. 1 shows the model that uses regional standard uptake value ratios (SUVR) and a target of binary class of tau positivity for prediction of THETA. This model was applied to predict tau status on each followup scan. Slopes of the meta‐ROIs’ tau SUVRs and THETA were evaluated by diagnostic group and as a function of baseline amyloid SUVR in discordant CU participants (where meta‐ROI and visual assessments did not match at baseline) and in incident‐THETA+ CU participants (whose THETA moved from below to above 1 over serial tau‐PET scans). We evaluated tau measurements as a function of Braak stages for neurofibrillary tangles.

**Result:**

Longitudinal plots of meta‐ROIs for diagnostic groups were very similar, but the expanded range of THETA based on visual positivity prediction clearly identified tau positive or tau‐negative scans. In discordant‐CU (n = 97) and incident‐THETA+ CU participants (n = 14 all amyloid positive at follow‐up but only 65% at baseline), the relationship between baseline amyloid and rate of tau increase was stronger for THETA than temporal meta‐ROI (Fig. 2). Separation between baseline and follow‐up was greater for THETA (t‐statistics = 90) compared to temporal meta‐ROI (t‐statistics = 20) (p<0.01) in the incident‐THETA+ CU participants. THETA showed a slightly stronger association with Braak stage than meta‐ROIs (rho = 0.87 vs. ≤0.83, p<0.05), with better separation of clinical diagnoses (Fig. 3).

**Conclusion:**

THETA remained clearly negative or positive in MCI and AD, providing consistent information on underlying etiology of impairment at both baseline and follow‐up. Although binary in its construction, THETA both provided separation of values based on tau status and its change correlated with baseline amyloid burden especially in discordant‐CU where tau deposition is not in typical meta‐ROIs. Further work is needed to confirm if THETA captures early tau changes.